# The relationship between prevalence and severity of lower urinary tract symptoms (LUTS), and body mass index and mid-abdominal circumference in men in a resource-poor community in Southeast Nigeria: a cross-sectional survey

**DOI:** 10.1186/s12894-019-0444-x

**Published:** 2019-02-21

**Authors:** Ikenna I. Nnabugwu, Fredrick O. Ugwumba, Emeka I. Udeh, Solomon K. Anyimba, Louis T. Okolie

**Affiliations:** 1 Department of Surgery, College of Medicine, University of Nigeria Ituku-Ozalla, KM 20 Enugu-PortHarcourt Highway, Enugu, PMB 01129 Nigeria; 2 Department of Health Administration and Management, University of Nigeria Enugu Campus, Enugu, Nigeria; 3 Department of Surgery, University of Nigeria Teaching Hospital Ituku-Ozalla, Enugu, Nigeria

**Keywords:** Men, LUTS, BMI, Mid-abdominal circumference

## Abstract

**Background:**

There is paucity of information on the community-based prevalence and severity of lower urinary tract symptoms (LUTS) in men who are 40 years and older in the southeast region of Nigeria. This study seeks to determine the community-based prevalence of LUTS and the relationship between LUTS, and body mass index (BMI) and mid-abdominal circumference (MAC) in men.

**Methods:**

An interviewer-administered, questionnaire-based survey. Three of nine settlement clusters were randomly selected while systematic random sampling of 1 in 3 eligible subjects was used to select participants. Analysis was done using SPSS® version 20.

**Results:**

One thousand three hundred and nineteen duly completed questionnaires were analyzed. The respondents are within ages 40-92 years with mean age 54.2 ± 10.2 years, mean BMI 25.97 ± 4.18Kg/m^2^ and mean MAC 89.80 ± 12.43 cm. Overall prevalence of LUTS is 20.2%. Nocturia at a prevalence of 19.2% is the most prevalent lower urinary tract symptom and also the earliest to manifest. LUTS prevalence and severity increases with increasing age. About 9.6% report moderate LUTS while 2.3% report severe LUTS. Storage LUTS are reported more frequently than voiding LUTS. LUTS did not vary significantly with BMI, MAC or Wealth-Index.

**Conclusion:**

LUTS prevalence and severity vary with age, but not with BMI, MAC or Wealth-Index.

## Background

Generally, the prevalence of lower urinary tract symptoms (LUTS) increases with increasing age in men. This may partly be as a result of increasing bladder outlet resistance consequent upon an enlarging prostate and partly due to other dysfunctional states of the lower urinary tract [1, 2]. Though the prevalence of LUTS among men may vary from community to community [3, 4], the onset of these symptoms is usually insidious and the course usually progressive.

Notably, the severity of these lower urinary tract symptoms tend to worsen over time from mild less bothersome symptoms to severe quite bothersome symptoms [5], though there may be intermittent remissions in symptoms of varying durations within this natural history of symptom progression. At any point in the course of LUTS, the International Prostate Symptom Score (IPSS) is recognized globally as a tool for assessing LUTS severity especially LUTS due to benign prostate enlargement [6].

Hospital-based estimates of the prevalence of LUTS in southeast Nigeria, a higher low-income country, has been documented [7], but community-based prevalence of LUTS in the region is essentially lacking. And figures from other resource-poor settings suggest that there could be wide variations between hospital-based prevalence and community-based prevalence of LUTS within a community [8]. There are indications as well that storage LUTS may be more prevalent than voiding LUTS and post-void LUTS [4].

Similarly, studies have suggested some association between LUTS in men and recognized indices of obesity such as body mass index (BMI) [9], abdominal circumference, and serum lipid parameters [4]. Arguably, higher BMI may be associated with higher income societies [10], and within a community, higher BMI may be prevalent among men with higher wealth-index [11]. However, there are no indications that a similar relationship is obtainable between LUTS and higher income or wealth-index.

As increasing proportions of the global population age due to improving living conditions and healthcare services, and with estimates indicating that Africa will be one of the continents with greatest increase in LUTS in the coming years [12], a firsthand knowledge of the prevalence of LUTS and its relationship with body mass index (BMI) among men in communities in Africa is relevant for planning.

Therefore, this study aims to provide the community-based prevalence of LUTS in an aging population of men in a resource-poor community in southeast Nigeria, and to identify the nature of the relationship between LUTS, and BMI and mid-abdominal circumference (MAC) among these men.

## Methods

An interviewer-administered quantitative questionnaire-based survey was conducted from March 5th to May 25th, 2018 in Enugu, the regional capital of southeast Nigeria with an estimated population of 983,000 persons, 22.0% of which are men who are 40 years and above, and are mostly civil servants and regional businessmen [13].

The first section of the survey questionnaire captured the age, weight, height and mid-abdominal circumference of the respondent, the second section was the validated International Prostate Symptom Score (IPSS) chart [14], and the thirdsection, adapted from Nigeria-General Household Survey, Panel 2015–2016, Wave 3 [15], assessed household living conditions and durable assets from which wealth indices were computed for individual respondents.

Face and construct validity of the study questionnaire were confirmed by experts in the field while reliability test yielded Cronbach’s α = 0.735 for the third section of the questionnaire. The questionnaires were administered by pre-tutored trainee surgeons and intern doctors.

A sample size of 372 was worked out using the formula $n=\frac{Z^2P\;\left(1-P\right)}{d^2}$where *P* is 0.591 [16]; *Z* is 1.96 and *d* is 0.05. An additional 10% of calculated sample size was included to cater for possible errors in data collection bringing the estimated minimum number of participants to 410.

Three of nine settlement clusters were randomly selected in Enugu urban for the study. Within the clusters, the study participants were selected through a systematic random sampling technique of 1 in every 3 eligible men. A minimum number of 410 participants was expected from each of the 3 selected clusters. Men dwelling in the communities who were 40 years and older were recruited. Written informed consent was obtained from each eligible participant before questionnaire was administered. Men who are known Diabetics, those who have spinal injury, urethral injury/stricture or cerebrovascular accident, and those on known diuretic drugs were excluded.

Simple frequency was used to determine prevalence of LUTS. Independent t test was used for comparison of means of BMI and MAC of men reporting LUTS against those of men reporting no LUTS. Principal Component Analysis (PCA) was used to create wealth indices using household durable assets and living conditions. Binary Logistic Regression Analysis was used to evaluate associations between the predictor variables (age, BMI, MAC, wealth index) and the dependent variable (presence of LUTS). Significance was set at *p* < 0.05. All analyses were done with SPSS® version 20. The University of Nigeria Teaching Hospital Bioethics Committee approved of the study.

## Results

Of the 1337 questionnaires administered and retrieved during the study, 1319 (98.6%) questionnaires were adequately completed and therefore could be analyzed. The socio-demographic characteristics of respondents are shown in Table 1.

**Table 1:** Shows the socio-demographic characteristics of respondents (*N* = 1319)

Variables	Frequency (%)
Age of Respondents (years)	
40–49	500 (37.9%)
50–59	420 (31.8%)
60–69	277 (21.0%)
70–79	110 (8.3%)
80–89	10 (0.8%)
90–99	2 (0.2%)
Body Mass Index (BMI) Kg/m^2^	
15.00–19.99	79 (6.0%)
20.00–24.99	522 (39.6%)
25.00–29.99	522 (39.6%)
30.00–34.99	158 (12.0%)
35.00–39.99	26 (2%)
40.00–44.99	11 (0.8%)
45.00–50.99	1 (0.1%)
Mid-Abd Circumference (cm)	
50.00–69.00	52 (3.9%)
70.00–89.00	617 (46.8%)
90.00–109.00	564 (42.8%)
110.00–129.00	85 (6.4%)
130.00–149.00	1 (0.1%)
Formal Education	
≤ 6 years	466 (35.3%)
>6 years	853 (64.7%)

Table 1 reveals that 90.7% of the respondents are below the age of 70 years, 39.6% are overweight while 14.9% are obese. Majority of the respondents (89.6%) have MAC between 70 cm and 109 cm about 64.7% had formal education beyond the primary level. The mean age of respondents is 54.2 ± 10.2 years, the mean BMI is 26.0 ± 4.18 kg/m^2^, the mean MAC is 89.80 ± 12.43 cm and the mean duration of formal education is 10.5 ± 5.3 years

Two hundred and sixty-seven respondents reported at least one lower urinary tract symptom giving a crude LUTS prevalence rate of 20.2% from this survey. Among these 267 respondents, 111 (41.6%) reported mild LUTS (IPSS 1–7), 126 (47.2%) reported moderate LUTS while 30 (11.2%) reported severe LUTS.

**Fig. 1:**
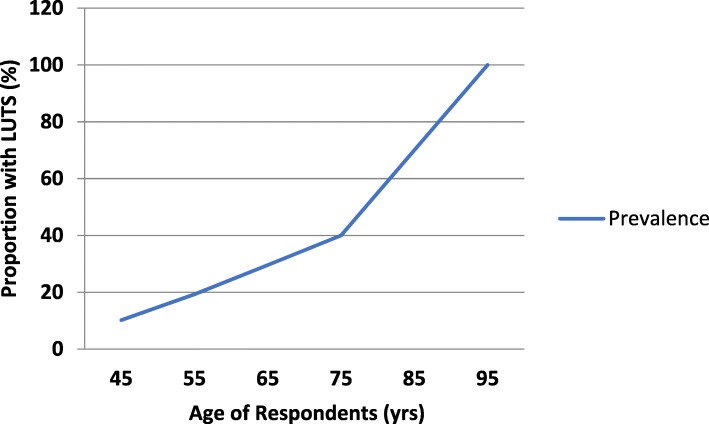
A line chart showing the relationship between respondents’ age and the proportion of respondents experiencing at least one lower urinary tract symptom (*N* = 1319). This figure shows that there is a gradual increase in the prevalence of LUTS from 40 years of age. The increase is at the rate of 7% per decade from 40 to 70 years. Beyond 70 years of age the rate of increase is nearly tripled

**Fig. 2:**
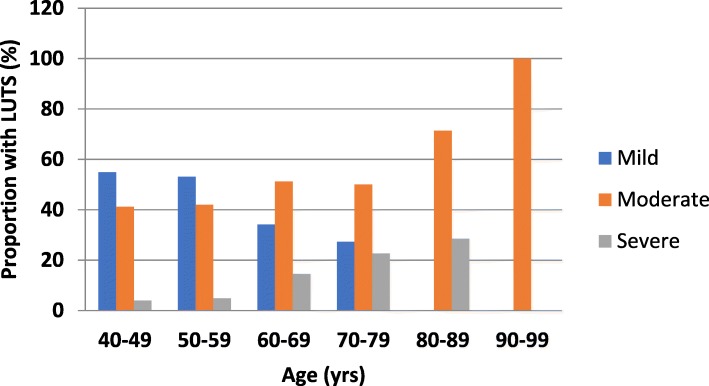
A cluster bar chart showing severity of lower urinary tract symptoms within the various age categories of respondents with LUTS (*N* = 267) Before the age of 60 years, mild LUTS is more prevalent, while beyond the age of 70 years, moderate to severe LUTS become more prevalent. This pattern is significant statistically

Sorting the respondents according to age reveals that the prevalence of LUTS from 40 years of age increases with increasing age as shown in Fig. 1. Similarly, the prevalence of moderate to severe LUTS increases with age (r 0.368; *p* < 0.001; Fig. 2) and there exists a positive correlation between LUTS severity and LUTS quality of life index (r 0.586; p < 0.001).

The bar chart in Fig. 2 displays the severity of LUTS according to age: the prevalence of moderate to severe LUTS increases with age while that of mild LUTS decreases with age (χ^2^ 28.421; df 10; p 0.002).

The body mass index (BMI) and the mid abdominal circumference (MAC) of respondents experiencing LUTS are compared against those of respondents who do not experience any LUTS using independent t test. There is no evidence (p 0.35) of difference in mean BMI of respondents experiencing LUTS (26.18 ± 3.99Kg/m^2^) and of those not experiencing LUTS (25.91 ± 4.23Kg/m^2^). There is also weak evidence, if any, (p 0.04) of a difference in mean MAC of respondents experiencing LUTS (90.20 ± 13.31 cm) and of those who are not experiencing LUTS (89.45 ± 12.18 cm). In addition, crosstab analysis showed no evidence of any association between men reporting LUTS and wealth-index (χ^2^ 6.22; p 0.18). Essentially therefore, there is no difference in the body sizes and the wealth-index of the 2 groups. Further analysis shows no evidence as well of correlation between LUTS severity and BMI (r 0.01; p 0.86), or between LUTS severity and MAC (r − 0.09; p 0.13). However, there is strong evidence of significant association between LUTS and age of respondents (χ^2^ 96.32; *p* < 0.001).

Table 2 shows that from the age of 40 years among men in this community, the odds that an older man will experience LUTS compared to a younger man is estimated to be 1.065 (95%CI 1.051–1.080).

**Table 2:** The outcome of Binary Logistic Regression analysis of factors relating with presence or not of LUTS

Variable	OR	95% CI		
		Lower Limit	Upper Limit	*p* value
Age of Respondent	1.065	1.051	1.080	< 0.001
BMI	1.007	0.969	1.046	0.732
MAC	1.010	0.997	1.022	0.139
Wealth Index	0.909	0.821	1.006	0.066

[*OR* Odds Ratio, *CI* Confidence Interval, *BMI* Body Mass Index, *MAC* Mid-Abdominal Circumference]

There is no evidence from this table, that the obesity indices BMI and MAC and the wealth index significantly influence LUTS in men, controlling for age

## Discussion

Majority of the participants in this study are between the ages of 40 and 70 years, had more than 6 years of formal education and are still actively involved in some form of economic activity (Table 1). The overall prevalence of LUTS from this study is 20.2% while the prevalence of moderate to severe LUTS is 11.9% (Table 3). These values are lower than the prevalence rates from the studies by Ojewola et al. [16], Olapade-Olaopa et al. [17] in southwest Nigeria and Chokkalingam et al. [18] in Accra, Ghana. The difference in prevalence rates may be partly explained by the observation that the study population in this study is younger than those in the other studies. At these observed prevalence rates, LUTS deserve some attention as a non-communicable disorder of importance in public health discussions in Nigeria. This is especially so in view of the existing misconceptions about LUTS in men, the significant delay in seeking medical care, and the attendant consequences thereof [19]. Commonly highlighted non-communicable chronic diseases such as systemic hypertension, Diabetes Mellitus, osteoarthritis, have similar prevalence rates, outcomes of management and consequences of neglect [20] compared to LUTS in men, and yet LUTS in men appear to receive less attention. Akin to many other studies [16], this study observes that storage LUTS are more prevalent than voiding LUTS, and nocturia is the most prevalent lower urinary tract symptom.

**Table 3:** Shows the frequency distribution of the various lower urinary tract symptoms among all respondents (*N* = 1319)

Variables	Frequency (n)	Proportion (%)
Frequency of urination	180	13.6%
Urgency	175	13.3%
Nocturia ≥ 1 per night	253	19.2%
Nocturia ≥ 2 per night	229	17.4%
At Least 1 Storage Symptom with Nocturia ≥ 1	263	19.9%
At Least 1 Storage Symptom with Nocturia ≥ 2	262	19.9%
Weak Stream	131	9.9%
Intermittency	104	7.9%
Straining	109	8.3%
Incomplete Emptying	125	9.4%
At Least 1 Voiding Symptom	181	13.7%
Any Lower Urinary Tract Symptom	**267**	**20.2%**
Mild LUTS (IPSS 1–7)	111	8.4%
Moderate LUTS (IPSS 8–19)	126	9.6%
Severe LUTS (IPSS 20–35)	30	2.3%

[*LUTS* lower urinary tract symptoms]

The table indicates that nocturia defined as any void during night sleep or as greater than 1 void during night sleep is the most prevalent lower urinary tract symptom; storage LUTS are generally more prevalent than voiding LUTS and 11.9% reported moderate to severe LUTS

The survey also shows succinctly that the prevalence rates of LUTS increases with advancing age (Fig. 1) similar to reports from other studies [21, 22] In addition, the severity of LUTS significantly worsens with advancing age (χ^2^ 28.421; df 10; p 0.002; Fig. 2). These findings are in keeping with the recognized progressive nature of the chronic symptom complex [23]., Though remissions in symptoms of various durations can, and do, occur during the natural course of these lower urinary tract symptoms [24], the aggregate outlook is usually that the symptoms gradually worsen, or at the best, persist as age progresses. The odds that an older man from the age of 40 years in the community will report LUTS, controlling for BMI, MAC and higher wealth index, is 1.065 (*p* < 0.001) (Table 2).

Though there is very weak evidence of any significant difference in MAC of men reporting LUTS and those reporting no LUTS (t − 2.057; df 1317; p 0.04), there exists no evidence of any significant difference in BMI of the 2 groups (t 0.938; df 1317; p 0.35). Similar studies in southwest Nigeria [25], in Korea Republic [26], in Italy [27] and in USA [5] report, as well, the absence of any significant difference in LUTS among men of various anthropometric dimensions. The explanation for this may be that while LUTS occurrence and progression are related to aging, BMI and MAC are not consistent in their relationship with aging. On the contrary, some other studies have reported significant positive correlation [28] or negative correlation [29] between LUTS and BMI as well as other components of metabolic syndrome. These conflicting observations suggest that there may be no direct causal relationship between LUTS, BMI, MAC and other metabolic syndrome components. Such absence of a significant correlation between these variables is the conclusion from a recent review of evidences from available studies by Sebastianelli & Gacci [30].

## Conclusions

This survey reveals that the overall prevalence rate of LUTS among men 40 years and older in this community is 20.2% with 58.4% of them experiencing moderate to severe LUTS. LUTS prevalence and severity increases with increasing age, but not with increasing obesity indices, BMI and MAC, or with increasing wealth-index. These observations suggest that attention should be given to the disorder during public health discuss. In addition, the observations contribute knowledge to the nature of the relationship between LUTS, aging, obesity and affluence in black African men in Nigeria,

## Abbreviations

BMI: Body Mass Index
IPSS: International Prostate Symptom Score
LUTS: Lower Urinary Tract Symptoms
MAC: Mid-abdominal Circumference
